# Complete-genome sequencing elucidates outbreak dynamics of CA-MRSA USA300 (ST8-*spa* t008) in an academic hospital of Paramaribo, Republic of Suriname

**DOI:** 10.1038/srep41050

**Published:** 2017-01-20

**Authors:** Artur J. Sabat, Sandra M. Hermelijn, Viktoria Akkerboom, Amadu Juliana, John E. Degener, Hajo Grundmann, Alexander W. Friedrich

**Affiliations:** 1 Department of Medical Microbiology, University of Groningen, University Medical Center Groningen, Groningen, The Netherlands; 2 Medical Microbiology Laboratory, Academic Hospital Paramaribo, Republic of Suriname; 3 Department of Pediatrics, Academic Hospital Paramaribo, Republic of Suriname; 4 Department of Infection Prevention and Hospital Hygiene, Faculty of Medicine, University of Freiburg, Freiburg, Germany

## Abstract

We report the investigation of an outbreak situation of methicillin-resistant *Staphylococcus aureus* (MRSA) that occurred at the Academic Hospital Paramaribo (AZP) in the Republic of Suriname from April to May 2013. We performed whole genome sequencing with complete gap closure for chromosomes and plasmids on all isolates. The outbreak involved 12 patients and 1 healthcare worker/nurse at the AZP. In total 24 isolates were investigated. *spa* typing, genome-wide single nucleotide polymorphism (SNP) analysis, *ad hoc* whole genome multilocus sequence typing (wgMLST), stable core genome MLST (cgMLST) and *in silico* PFGE were used to determine phylogenetic relatedness and to identify transmission. Whole-genome sequencing (WGS) showed that all isolates were members of genomic variants of the North American USA300 clone. However, WGS revealed a heterogeneous population structure of USA300 circulating at the AZP. We observed up to 8 SNPs or up to 5 alleles of difference by wgMLST when the isolates were recovered from different body sites of the same patient or if direct transmission between patients was most likely. This work describes the usefulness of complete genome sequencing of bacterial chromosomes and plasmids providing an unprecedented level of detail during outbreak investigations not being visible by using conventional typing methods.

Many human infections are caused by multi-drug resistant bacteria and methicillin-resistant *Staphylococcus aureus* (MRSA) is one of the most prevalent pathogens in hospitals worldwide. MRSA is easily transmitted in the health care setting and represents an ubiquitous problem wherever studies have been carried out. MRSA colonization is a significant risk factor for later infection^1^,^2^,^3^. Individuals, who acquire MRSA colonization during a hospital stay, also serve as reservoirs for further dissemination in the healthcare settings as well as in the community. The prevention of nosocomial MRSA infections, especially in intensive care units with seriously ill patients, primarily include improved hand hygiene practices, extensive cleaning and disinfection of the hospital environment, and screening for MRSA carriage followed by isolation of documented carriers.

Accurate typing methods are required for efficient infection control. In outbreak settings, a typing method should have a high discriminatory power allowing correct exclusion of epidemiologically unrelated isolates. Moreover, typing methods used for epidemiological investigations should also be able to discern between genetically very closely related isolates, thus improving the resolution to near forensic precision. At the same time, methods should be simple, inexpensive, rapid, highly reproducible and results easily interpretable^4^. To date, many different molecular typing methods for discriminating MRSA isolates have been developed. For MRSA outbreaks, conventional molecular typing methods such as pulsed-field gel electrophoresis (PFGE), *spa* typing or multiple locus variable number tandem repeat analysis (MLVA) have been widely used before the advent of the whole genome sequencing (WGS) era^5^,^6^,^7^,^8^,^9^. However, conventional typing methods appear not to have appropriate discriminatory power to show whether an outbreak in a hospital resulted from multiple independent introductions of a predominant MRSA lineage from a wider population or from a single source.

Next generation sequencing (NGS) technologies including genome-wide analysis tools have recently been developed for routine typing of bacterial pathogens at clinically relevant timescales and at cost-benefit appropriate price levels. To date analysis of complete genomes has the ultimate resolution power enabling the detection of single point mutations (single nucleotide polymorphisms, SNPs) as well as the presence or absence of mobile genetic elements and genomic data have become increasingly available for identification of bacterial transmission between patients during outbreak investigations^10^,^11^,^12^,^13^,^14^,^15^,^16^. In this study, we obtained complete genome sequences of 24 MRSA isolates recovered from patients during an outbreak in an academic hospital in Paramaribo in the Republic of Suriname. Together with epidemiological data, we assessed the possible transmission events among 12 patients and 1 healthcare worker/nurse and within-host and between-host genetic diversity of MRSA isolates.

## Results

### Description of the outbreak

The outbreak occurred at the Academic Hospital Paramaribo (AZP) in the Republic of Suriname, a small middle income country in South America with a population of roughly half a million people. AZP is the largest, most advanced and only tertiary hospital in Suriname. In 2013 it had 504 beds (including 34 beds in a “Sanatorium”, which is at another location than the hospital), 23564 total inpatient admissions and 243721 outpatient visits. The staff consisted of 1110 healthcare, support and clerical staff. When the outbreak occurred, an investigation was triggered by the infection control team immediately and control measures were implemented. Patients who were in the same room as a positive case (n = 31) were screened using nasal, pharyngeal and perineum swabs and swabs of insert places of lines, if present. Healthcare workers who had contact with a MRSA patient (n = 111) were screened using nasal and pharyngeal swabs. Using these interventions the outbreak was halted within a short time and the MRSA isolation at the AZP returned to the background rate (around 1%). A total of 24 MRSA isolates were investigated in this study. Twelve patients and 1 health care worker/nurse were infected and/or colonized with MRSA at the AZP between 24th of April and 28th of May 2013 (Table 1). In some cases, multiple isolates were recovered from different body sites of single patients. The nurse worked in the ICU at the AZP for short period of time at the time of admission of patient 3 but went back to a district hospital about 230 km from the AZP where she was screened. Additionally, 2 MRSA isolates, recovered from a child (patient 1) hospitalized at the AZP one month before the outbreak was recognized, were used in this study.

### Molecular characterization

*spa* typing assigned *spa* type t008 to all of isolates. From the WGS data, *in silico* MLST identified all the isolates belonged to single sequence type (ST) 8, and that they were clonally related to ST8-*spa* t008 isolates belonging to USA300. Moreover, all isolates were positive for arginine catabolic mobile element (ACME) type I and Panton-Valentine leukocidin (PVL).

### SNP analysis

**Figure 1:**
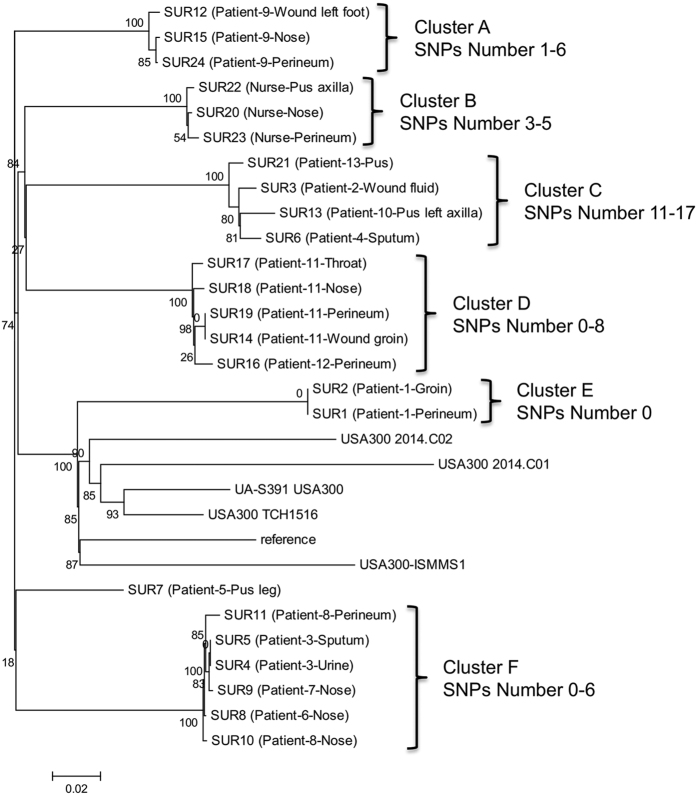
Phylogenetic relationship of outbreak isolates from AZP in 2013 mapped against reference type strain USA300 FPR3757. Phylogenetic maximum likelihood tree constructed on the basis of SNPs was obtained by CSI phylogeny 1.4 (https://cge.cbs.dtu.dk/services/CSIPhylogeny/). A confidence score ranging from 0 to 1 (x100 during visualization in MEGA6 software) was calculated for robustness evaluation of the nodes. The scale bar indicates the evolutionary distance between the sequences determined by 0.02 substitutions per nucleotide at the variable positions.

Thirty complete chromosome sequences (representing 24 isolates from Suriname and 6 fully annotated USA300 outgroup genomes) were subjected to the CSI Phylogeny 1.4 server in order to investigate their SNP-based phylogeny. The sequences were aligned to the reference chromosome (isolate USA300 FPR3757) and then SNPs were called. The reference chromosome was 2,872,769 nucleotides in size and 2,837,117 positions (98.76% of reference chromosome) were found in all analyzed chromosomes. The CSI Phylogeny 1.4 revealed 6 clusters (A to F) of very closely related isolates (Fig. 1). There were seven unique chromosome sequences that did not group with any of the six clusters. These included the six USA300 outgroup genomes, and one isolate (Sur7) distantly related to cluster F that originated from a single patient (patient 5). Maximal intra-cluster diversity was 17 SNPs (between Patient-10-Pus left axilla and Patient-13-Pus) whereas minimal inter-cluster diversity was 69 SNPs (between Patient-5-Pus leg and Patient-9-Perineum). Clusters that included isolates from epidemiologically associated patients, i.e. patients that were either treated in the same ward or the same room during overlapping admission periods (Table 1) differed by no more than 8 SNPs. No more than 6 SNPs could be ascertained for repeat isolates from different anatomical sites of same patients and the nurse. There were three pairs of isolates, one in each of the clusters D, E and F that were indistinguishable on the basis of whole genome analysis, and invariably they were isolated from the same patients. We, however, also found that the within cluster SNP distances could not always discriminate between same patient or different patient origin. This can be illustrated in cluster F where the nose and perineal isolate from patient 8 differed by five SNPs but by only one to three SNPs from isolates of other patients. Sequences within cluster C differed by 11 to 17 SNPs, representing the most heterogeneous of all clusters. Isolates within this cluster were from four different patients who were treated in different wards and direct routes of transmission appeared unlikely on epidemiological grounds.

### *Ad hoc* wgMLST and stable cgMLST comparisons

**Figure 2:**
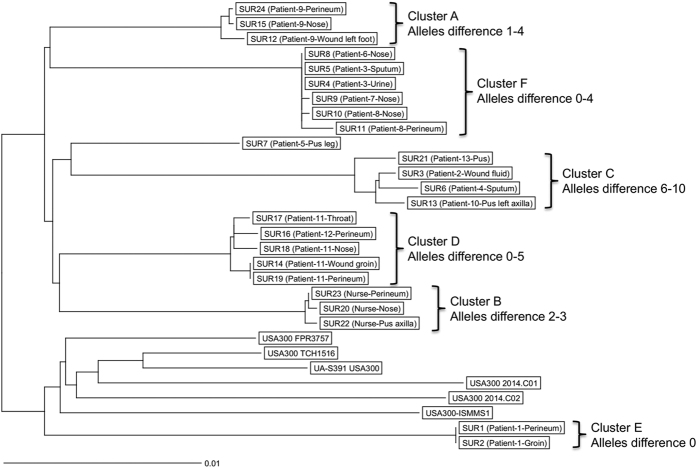
A dendrogram based on the *ad hoc* wgMLST approach. The allele-based Neighbor Joining tree was built using SeqSphere+ software. Clusters designation according to the genomic analysis in Fig. 1.

To compare core SNP phylogenies with gene-by-gene approaches, we applied an *ad hoc* wgMLST, which utilized the genetically most related reference genome, to enhance comparability by including a maximum of homologous coding sequences. In this way, not only the core but also most of the accessory genome can be compared. The complete chromosome of the *S. aureus* strain USA300 FPR3757 with 2,869 annotated genes, served as the reference for this *ad hoc* wgMLST analysis. Of all annotated genes, 2,767 were included for wgMLST by the SeqSphere software while 102 genes were excluded. The wgMLST-based partition (Fig. 2) was in perfect agreement with that of the SNP-based analysis (Fig. 1). The only difference was a slightly lower level of discrimination of wgMLST, whereby more isolates remained indistinguishable. Within wgMLST clusters, up to 10 alleles differences were observed, while unrelated isolates differed between each other by at least 48 alleles. For isolates recovered from the same patient or where direct transmission between patients was assumed (clusters A, B, D, E and F) the isolates differed by up to 5 alleles. Isolates of cluster C where no transmission was plausible differed by 6 to 10 alleles.

**Figure 3:**
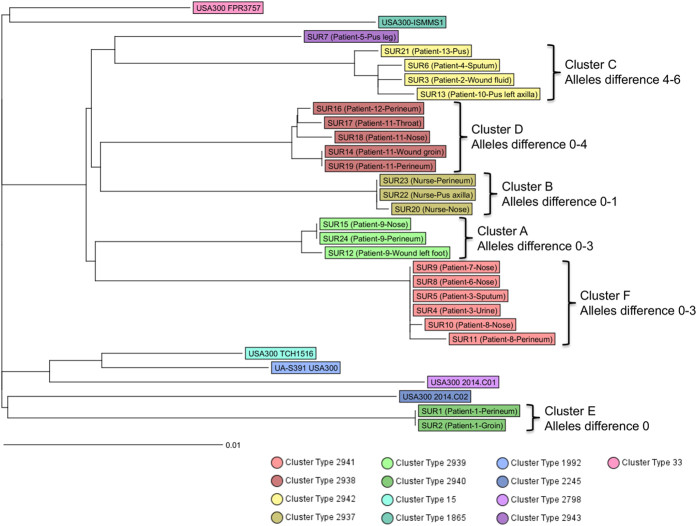
A dendrogram based on the cgMLST approach. The allele-based Neighbor Joining tree was built using SeqSphere+ software. Clusters designation according to the SNPs analysis. Cluster types in the dendrogram are indicated by the colors.

In contrast to *ad hoc* wgMLST, the stable cgMLST approach, which utilizes always the same reference genome and only core genome genes, represents a species-wide tool for genomic comparison providing a public expandable universal nomenclature. We applied stable cgMLST to identify cluster types facilitating future studies and global data exchange. For this approach *S. aureus* COL type strain provided the reference and a standard set of 1,861 genes for gene-by-gene comparison is used. As the number of genes is roughly 900 genes fewer than that of wgMLST (1,861 vs 2,767, respectively) discriminatory ability of cgMLST analysis does not match that of SNP- or wgMLST-based approaches (Fig. 3). Nonetheless, the overall partition remained the same. We found 4 pairs and a single four of identical isolates using cgMLST. The isolates differed by up to 6 alleles within a cluster or by at least 36 alleles when unrelated isolates were compared. The analysis of the 30 USA300 isolates revealed 13 cluster types, which are specified in Fig. 3.

### *In silico* PFGE cluster analysis

**Figure 4:**
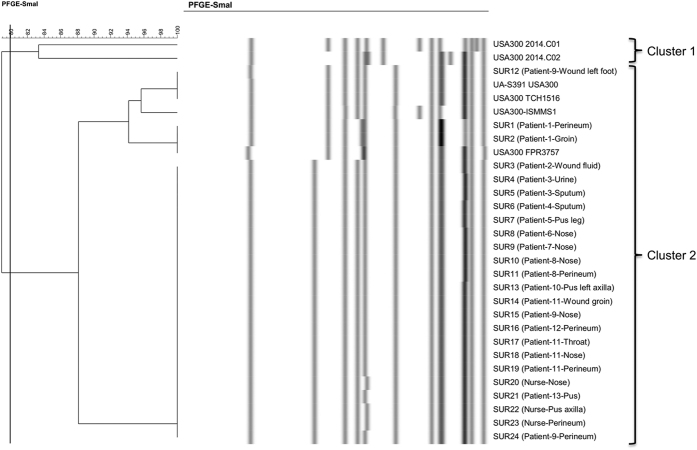
*In silico* prediction of PFGE *Sma*I fragment pattern. Dendrogram generated by the UPGMA algorithm using BioNumerics software. Clusters were grouped at an 80% similarity cutoff value.

PFGE has for long been considered as the ‘gold standard’ among molecular typing methods for *S. aureus* and is still widely used^4^. We wanted to establish if a prediction of PFGE *Sma*I-restriction fragment patterns based on *in silico* analysis could resolve the same clusters as SNP-based and wgMLST-based methods. Overall, restriction profiles showed a low degree of polymorphism among all isolates. Our *in silico* analysis produced only 6 *Sma*I-restriction patterns for all 30 USA300 isolates, which were classified into 2 distinct clusters based on the similarity cutoff of 80% (Fig. 4). Cluster 1 was composed of 2 non-Suriname isolates. All USA300 Suriname isolates were related with each other and belonged to the cluster 2. The majority of USA300 isolates from Suriname (n = 21) shared an identical PFGE pattern, whereas the same isolates were divided into 5 different clusters and a single unique isolate by the SNP and wgMLST. Moreover, 2 isolates from patient 1 and one isolate from patient 9 were indistinguishable by *in silico Sma*I-macrorestriction analysis from epidemiologically unrelated USA300 reference genomes. These shared the same pattern with USA300 FPR3757, and UA-S391 USA300 and USA300 TCH1516, respectively.

### Genetic diversity of the chromosomes among Suriname USA300 isolates

**Figure 5:**
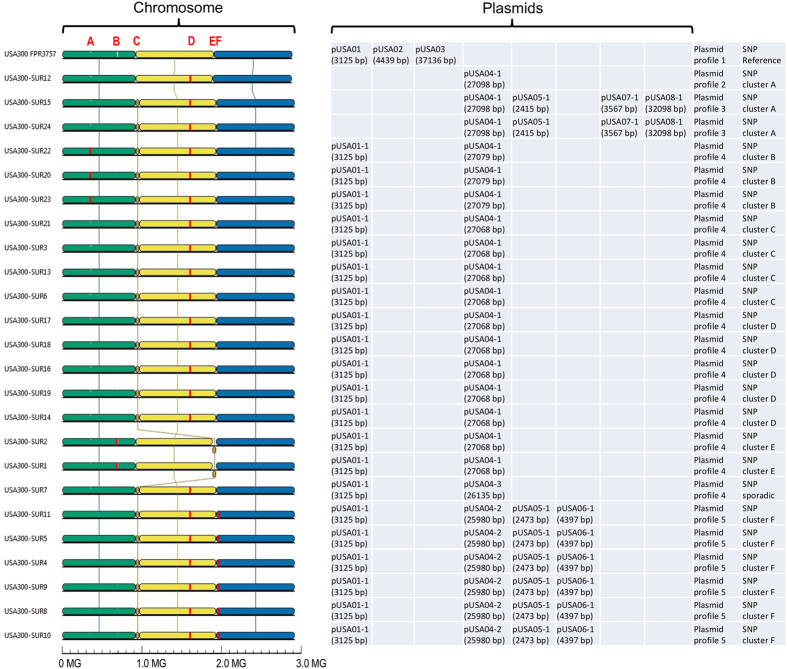
Diversity of accessory genomes among the USA300 isolates from patients in Suriname compared to the reference FPR3757. Transverse red lines inside of the chromosomes indicate sites for a genomic deletion or insertion: (A) deletion of 4.5 kb fragment (FPR3757, positions 347431..351951); (B) deletion of 12.1 kb fragment from a mobile element (FPR3757, positions 1631620..1643721); (C) insertion of a new phage phiUSA300-2 (FPR3757, position 903353..903354); (D) insertion of 3.3 kb fragment (FPR3757, position 1550714..1550715); (E) insertion of a new phage phiUSA300-1 (FPR3757, position 1883465. 1883466); (F) deletion of 1.7 kb fragment (FPR3757, positions 1939480..1941223). A scale at the bottom indicates the size of the chromosomes. The sizes of plasmids are indicated in brackets.

The complete sequences (closed chromosomes) of isolates from Suriname consisted of 2,876,067–2,919,595 bp. Compared to the reference USA300 FPR3757 chromosome, we found 2 gene insertions and 3 gene deletions (Fig. 5). The most striking feature of these isolates was a presence of 2 new phages (Fig. 5. positions C and E; Table S1), designated phiUSA300-1 and phiUSA300-2, integrated into the chromosome with the exception of the isolate SUR12. The 2 new phages had different chromosomal integration sites and were inserted in opposite orientation (Fig. 5, positions C and E). While phage phiUSA300-1 (43.1 kb) was only found in the chromosome of isolates from cluster E, phage phiUSA300-2 (43.5 kb) was present in all other isolates from Surinamese patients. The nucleotide sequences of phage phiUSA300-1 were indistinguishable and the nucleotide sequences of phage phiUSA300-2 were nearly identical (99% sequence identity). Sequence analysis showed that phiUSA300-1 and phiUSA300-2 were moderately related (77%) and were most related to phage phiETA2 with 61% and 66% sequence identity, respectively. With the exception of cluster E, all other Suriname isolates had a 3.3 kb fragment containing the *dfrG* gene conferring trimethoprim resistance (Fig. 5, position D). Integration of this 3.3 kb fragment occurred inside of phage ϕSA2usa harboring *lukF*-*lukS* genes coding for PVL. In contrast to gene insertions, gene deletions were cluster specific. Cluster B contained deletion of 4.5 kb fragment encompassing 7 genes encoding hypothetical proteins (Fig. 5, position A). All Suriname isolates with the exception of cluster E (SUR1 and SUR2) had a mobile element of 13.4 kb in length (Fig. 5, position B), which also exists in the USA 300 FPR3757 genome at position 1630719-1644075. This element was translocated in the Surinamese isolates and inserted into the gene (FPR3757, locus tag SAUSA300_0606) encoding putative membrane protein. The central part of the mobile element of 12.1 kb in length was deleted from genomic DNA of two Suriname isolates representing cluster E. Cluster F had a deletion of 1.7 kb region resulting in a loss of the genes *splD* and *splE* encoding extracellular serine proteases, representing important virulence factors of *S. aureus* (Fig. 5, position F).

### Plasmid content

The USA300 isolates from Suriname tested in this study possessed between 1 to 4 plasmids (Fig. 5) generally harboring one large plasmid of 26 to 32 kb (except of SUR12) and at least one small plasmid of 2 to 4 kb. Plasmids recovered from Suriname USA300 isolates were designated in compliance with nomenclature for plasmids from the reference genome USA300 FPR3757, which possessed three plasmids, pUSA01, pUSA02 and pUSA03. When a plasmid from an isolate recovered in Suriname was not present in the genomic DNA of FPR3757, it received a consecutive number, starting from pUSA04. Surinamese isolates contained six different plasmid types and four different plasmid profiles (Fig. 5). Plasmid content was not always in agreement with the SNP analysis of chromosomes. The isolates of clusters C, D and E shared the same plasmids, while the isolates of cluster A, recovered from the same patient, had 2 different plasmid profiles.

**Figure 6:**
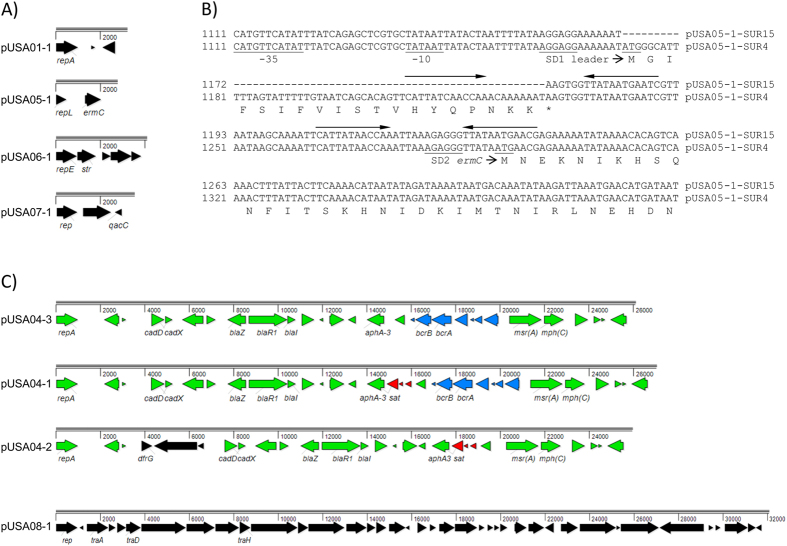
Genetic organization of Suriname USA300 plasmids. (**A**) Gene content of small plasmids (2–4 kb). Only the following selected genes are annotated: the determinant encoding replication initiation protein (*rep*); the determinant encoding macrolide resistance (*ermC*); the determinant encoding streptomycin resistance (*str*); the determinant encoding quaternary ammonium compound resistance efflux pump (*qacC*). (**B**) Comparison of the two different types of *ermC* regulatory regions. Plasmids pUSA05-1-SUR4 (representative of cluster F) and pUSA05-1-SUR15 (representative of cluster (**A**) represent the inducible and the constitutive type of *erm*C gene expression, respectively. The positions of the *ermC* promoter (−35 and −10) are indicated by underlining, as are the start of transcription of the leader peptide and *ermC* methylase gene and ribosome-binding sites SD1 and SD2. Amino acid sequences deduced from the nucleotide sequences are given in the single letter code. Asterisk indicates translational stop codon. Inverted repeated sequences are marked by arrows. (**C**) Gene content of large plasmids (≥26 kb). The genes common to all pUSA04 plasmids are shown in green. The genes common to plasmids pUSA04-1 and −3 are shown in blue. The genes common to plasmids pUSA04-1 and −2 are shown in red. The genes unique for a plasmid are shown in black. Only the following selected genes are annotated: the determinant encoding replication initiation protein (*rep*); the determinant encoding macrolide resistance (*ermC*); the determinant encoding streptomycin resistance (*str*); the determinant encoding cadmium resistance (*cadD*); the determinant encoding positive regulator of cadmium resistance (*cadX*); the determinant encoding beta-lactamase, penicillin resistance (*blaZ*); the determinant encoding positive regulator of penicillin resistance (*blaR1*); the determinant encoding negative regulator of penicillin resistance (*blaI*); the determinant encoding 3′5′-amino-glycoside phosphotransferase, neo-/kanamycin resistance (*aphA3*); the determinant encoding membrane-bound permease, bacitracin resistance (*bcrB*); the determinant encoding ATP-binding protein, bacitracin resistance (*bcrA*); the determinant encoding erythromycin exporter, macrolide, lincosamide and streptogramin B resistance (*msr(A*)); the determinant encoding phosphotransferase, macrolide resistance (*mph(C*)); the determinant encoding acetyltransferase, streptothricin resistance (*sat*); the determinant encoding dihydrofolate reductase, trimethoprim resistance (*dfrG*); the determinants encoding conjugal transfer elements (*traADH*).

With the exception of cluster A, all isolates harbored a small 3.1 kb plasmid (Figs 5 and 6A). The plasmid by sequence and size was identical to cryptic plasmid pUSA01 of strain USA300 FPR3757^17^ except isolate SUR7 in which one SNP was found in pUSA01-1. The isolates of clusters A and F possessed additional small plasmids (Fig. 5). A 2.4 kb plasmid (pUSA05-1) encoding resistance to erythromycin was found in both clusters (Fig. 6A). A comparison of the DNA sequences of pUSA05-1 showed that these sequences were identical within a cluster (A or F) but differed between the clusters by a 58-bp deletion in the 5′ regulatory region of *ermC* (Fig. 6B). This 58-bp deletion in isolates of cluster A comprised the entire reading frame of the leader peptide as well as one of the inverted repeated sequences. Previous investigations have shown that sequence changes in the regulatory region of *erm*C result in a switch from the inducible to the constitutive type of *erm*C gene expression^18^,^19^,^20^,^21^. The 2.4 kb plasmid pUSA05-1 was almost identical (99%) or shared 97% of identity to plasmid pWBG751 of strain ST1-MRSA-IVa, when isolates from clusters F or A were tested, respectively. A 4.4 kb plasmid (pUSA06-1) conferring resistance to streptomycin was found exclusively in isolates from cluster F (Figs 5 and 6A), while a 3.6 kb plasmid (pUSA07-1) was present only in the genome of isolates representing cluster A (Fig. 5). The 4.4 kb plasmid pUSA06-1 was nearly identical (99%) to the pS194 plasmid and was closely related to other members of the pT181 family. The 3.6 kb plasmid pUSA07-1 from cluster A was recombinant possessing the *qacC* gene encoding a quaternary ammonium compound resistance efflux pump (Fig. 6A).

All USA300 isolates from Suriname harbored a large plasmid (pUSA04) that in the majority of the clusters, from A to E (plasmid pUSA04-1), was essentially identical (27.068 kb, 99% sequence identity) to plasmid pUSA300HOUMR (27.041 kb, possessing genes conferring resistance to cadmium, bacitracin, macrolides, penicillin, kanamycin and streptothricin) (Figs 5 and 6C). Plasmids pUSA04 among Surinamese isolates shared 99% sequence identity but differed between each other by small deletions. When a deletion resulted in a loss of portion of the genes, the plasmid name received an additional digit, pUSA04-1, pUSA04-2 and pUSA04-3. The pUSA04-1 plasmids were identical or differed only by 1 SNP within a cluster, while between the clusters they differed by 4 to 7 SNPs. The isolate SUR7 variant of pUSA300HOUMR lacked the gene *sat* encoding streptothricin resistance (plasmid pUSA04-3) (Fig. 6C). The biggest rearrangements were found in plasmid pUSA04-2 of cluster F (Fig. 6C). Plasmid pUSA04-2 of cluster F was smaller by 900 bp than pUSA300HOUMR and did not contain the genes *bcrA* and *bcrB* conferring resistance to bacitracin but possessed the *dfrG* gene encoding trimethoprim resistance. The isolates SUR15 and SUR24 from cluster A were the only isolates that had a second large plasmid designated pUSA08-1 (Fig. 5). The plasmid pUSA08-1 was 32.098 kb in size, identical in SUR15 and SUR24 isolates but highly divergent from any nucleotide sequence deposited in the GenBank database. These plasmids possessed the *tra* genes encoding conjugative elements necessary for DNA transmission and no antimicrobial resistance gene (Fig. 6C).

### Antibiotic resistance

Phenotypic susceptibility testing showed that antibiotic resistance was not cluster specific. Isolates of clusters A, B, C, D and E and sporadic isolate SUR7 showed identical antibiotic resistance profiles (Table 2). Moreover, in the cluster A two isolates, SUR15 and SUR24, were resistant to clindamycin, while the isolate SUR12 from the same cluster was susceptible to this antibiotic. Among the 21 antibiotics tested, all Suriname USA300 isolates were resistant to beta-lactams (oxacillin, cefoxitin and penicillin), kanamycin, ciprofloxacin and erythromycin. Additionally, the isolates of cluster F showed resistance to rifampicin and streptomycin, and two isolates in cluster A were resistant to clindamycin.

We also determined the effectiveness of WGS as a tool for predicting antibiotic resistance. The antibiotic resistance mechanisms inferred from the genome sequences are specified in Table 2. For almost all antibiotics, WGS accurately predicted antimicrobial resistance. The *ermC* gene conferring resistance to clindamycin was found on plasmid pUSA05-1. This plasmid was identified in all isolates of cluster F and in two out of three isolates of cluster A. However, only the isolates of cluster A with the plasmid pUSA05-1 were resistant to clindamycin (Table 2). The deduced amino acid sequences of *ermC* were identical in both clusters. The difference was found in the promoter region, which showed the presence or the absence of the leader sequence (Fig. 6B). All constitutively expressed *erm*C genes in two isolates of cluster A revealed the 58-bp leader sequence deletion. To detect inducible clindamycin resistance, the D test method was performed using clindamycin and erythromycin disks. Phenotypic D test method revealed inducible resistance to clindamycin conferred by the plasmid pUSA05-1 among the isolates of cluster F.

## Discussion

We constructed a phylogenetic tree based on genome-wide SNP analysis using data for 30 USA300 MRSA isolates, including 24 field isolates recovered from patients and a nurse from the AZP in the Republic of Suriname and 6 unrelated reference genomes. Several distinct clusters and single branches were observed, which differed by at least 69 SNPs. Within a cluster isolates differed by up to 17 SNPs (Fig. 1). Isolates recovered from the same patient or when patient-to-patient transmission was likely, a SNP difference of ≤8 (which reflected ≤5 alleles of difference by wgMLST) was found but when no epidemiological association could be ascertained, isolates differed by 11 to 17 SNPs (and between 6 and 10 alleles by wgMLST). Previous studies suggested that, depending on *S. aureus* clonal complex, core genomes accumulate between 3–8 mutations per year^22^,^23^,^24^,^25^,^26^. Given the patients treatment intervals in the AZP, we can therefore assume that there were no direct transmissions of isolates for which 11–17 SNPs differences were established. Thus WGS analysis and epidemiological investigations can be mutually supportive.

Although all investigated isolates from Suriname belonged to a single *spa* type and the situation in the hospital in Paramaribo from April to May 2013 could be regarded by conventional typing approaches as a single big outbreak, the population structure of MRSA exhibited considerable diversity at the genomic level which was reflected by the SNPs, wgMLST and accessory genome analyses. It is therefore conceivable that the isolate clusters circulating in the hospital resulted from independent introductions from the wider USA300 population co-circulating in the community of the Republic of Suriname. Similar conclusions were drawn from previous studies which suggested that clonal pools of *S. aureus* can exist in the community that occasionally lead to infections and the admission of patients carrying closely related organisms that conventional typing methods would not correctly be able to discern^27^. Our results show that PFGE has a limited discriminatory power at a local scale, when a single lineage dominates in the population. Thus PFGE can no longer be regarded as the gold standard for outbreak investigations for MRSA.

Recent phylogenetic analyses^28^ revealed 2 dominant clades within the USA300 lineage that had a putative common ancestor in 1975 but segregated by geographical region, in North America (USA300 North American, USA300-NA) and in South America (USA300 Latin American variant, USA300-LV). The isolates recovered in Suriname carried the ACME element characteristic for the USA300-NA clade^28^. The isolates analyzed in this study also showed fluoroquinolone-resistance with the same mutations in *grlA* (S80Y) and *gyrA* (S84L) (Table 2) like those found in the USA300-NA clade, which has spread globally^10^. Moreover, all isolates from Suriname carried pUSA04 plasmid related to plasmid pUSA300HOUMR found in USA300 isolates recovered from patients in the United States. Genetic features such as the copper and mercury resistance (COMER) mobile element, which is characteristic for USA300-LV, was not found in the genomes of Suriname USA300 isolates. Despite the fact that USA300-LV reportedly spread through communities and hospital settings in Colombia, Venezuela and Ecuador, based on data obtained by this study, it appears that a subclade of USA300-NA to be the predominant clone circulating in the Republic of Suriname.

The *ad hoc* wgMLST results produced high-resolution allelic profiles, which were used to discriminate the MRSA isolates and to identify transmission chains among patients admitted at the AZP with nearly the same discriminatory ability as genome-wide SNP analysis. To develop the *ad hoc* wgMLST scheme we used the complete chromosome of the *S. aureus* strain USA300 FPR3757 as the reference which showed to be the genetically closest genome to those recovered from patients in Paramaribo. This approach ensures the highest possible discriminatory power among the MLST schemes because it utilizes the core, the core variable and the accessory genome from a highly related strain. The *ad hoc* schemes are very useful for local outbreak analyses but are not applicable for investigations of multiple outbreaks with different genetic backgrounds or for continuous monitoring. Moreover, it is not possible to share an allele nomenclature between laboratories when an *ad hoc* wgMLST scheme for local outbreak control has been developed. Therefore, we also used stable cgMLST, which in contrast to the *ad hoc* scheme always analyzes the same set of species-specific core genes. Although the cgMLST approach is endowed with a lower discrimination potential compared to *ad hoc* wgMLST, it facilitates standardization. This could be crucial in infection control to enable comparisons by providing expandable nomenclature, which can be universal for all laboratories. Moreover, the cgMLST method is better suited to compare analyzed isolates with stored data and for prospective analysis.

In the current study *in silico Sma*I restriction patterns were derived from complete genome sequences of 30 *S. aureus* USA300 isolates. Cluster analysis was performed with the BioNumerics software applying 0.5% optimization and 1% tolerance values, which allowed us to better simulate real PFGE on traditional agarose gels, which suffer from a lack of the resolution power to distinguish bands of nearly identical size (fragments differing from each other in size by less than 5–10%). Therefore, also in *in silico* PFGE analysis bands of nearly identical size were recognized as identical by the software. Even in perfectly standardized PFGE settings there are still technical problems that are inherent to the method^29^. In the *in silico* analysis we did not assess such parameters, which may influence the interpretation of conventional PFGE results like: (i) methylation of *Sma*I restriction sites; (ii) normalization of the PFGE gels due to the physical distance between the patterns being compared on a gel or on different gels; or (iii) misdetection of subtle pattern differences by the software and/or the human eye. These problems are not straightforward to address in the *in silico* analysis. An advantage of *in silico* PFGE analysis conducted in this study was that we could use for comparison the publically available whole-genome sequence data sets for the isolates, which we physically did not have in our collection. Although PFGE has been considered as the gold standard method for molecular epidemiological strain typing our results showed the need for a new gold standard approach for MRSA outbreak investigations. Our results are in agreement with previous investigations in which conventional PFGE on traditional agarose gels was compared to WGS in presumptive MRSA outbreaks^30^.

Addition of whole-genome sequencing to an infection-control investigation of a MRSA outbreak at the Academic Hospital Paramaribo allowed enhanced identification of an MRSA transmission networks. This study demonstrates that high resolution genome sequence analysis may not only support epidemiological associations among clinical MRSA isolates collected during outbreak investigations but may also reveal relationships among the isolates not previously resolved by conventional typing methods. It seems clear that WGS constitutes a new gold standard in the analysis of hospital outbreaks and can be used in infection control to prevent MRSA transmission as well as to predict resistance and virulence.

## Materials and Methods

### Phenotypic antibiotic susceptibility testing

Antibiotic resistance to 22 antibiotics was determined by Etests (bioMérieux). Following antibiotics were tested: bacitracin, ciprofloxacin, clindamycin, daptomycin, gentamicin, kanamycin, tobramycin, sulfamethoxazole/trimethoprim, minocycline, tetracycline, erythromycin, chloramphenicol, linezolid, fusidic acid, mupirocin, rifampicin, amikacin, oxacillin, cefoxitin, penicillin, streptomycin and vancomycin. Methicillin-sensitive *S. aureus* ATCC 29213 was used as a negative control. Results were interpreted according to EUCAST clinical breakpoints, version 6.0, valid since 2016-01-01. To detect inducible clindamycin resistance, D-test method was performed according to the EUCAST guidelines using clindamycin (2 μg) and erythromycin (15 μg) disks (Becton Dickinson).

### *spa* typing

The procedure was carried out as previously described^31^. The *spa* types were assigned using Ridom StaphType software version 2.2.1.

### WGS and data analyses

One colony forming unit from visibly pure culture of each clinical isolate was selected for WGS analysis. Genomic DNA was extracted using the DNeasy Blood and Tissue Kit (Qiagen) and purified DNA was quantified with a Qubit 2.0 Fluorometer (Life Technologies). A next-generation sequencing approach was performed on an Illumina MiSeq system with DNA fragment libraries prepared using a Nextera XT v3 kit (Illumina) according to the manufacturer’s protocol. The samples were sequenced to obtain a minimum coverage of 100-fold. The fastq files (Illumina MiSeq) with read length of 300 bases were *de novo* assembled into contigs using the DNASTAR SeqMan NGen assembler (version 12.1.0). The resulting contigs were ordered by Mauve Contig Mover^32^ and alignment of contigs end to end to find overlap between adjoining contigs was achieved using The DNASTAR SeqMan Pro software (version 12.1.0). The remaining gaps between contigs were closed by PCR amplification and Sanger sequencing. The assembly files with the complete and closed genomes were exported as fasta files and used in further analyses. Automated genome annotation was performed using the NCBI Prokaryotic Genome Annotation Pipeline (http://www.ncbi.nlm.nih.gov/genome/annotation_prok/). The DNA sequences were aligned using Mauve implemented in the DNASTAR MegAlign Pro software (version 13.0.0). Multilocus sequence typing (MLST) was performed using the Center for Genomic Epidemiology server online tool MLST 1.8 (https://cge.cbs.dtu.dk/services/MLST/)^33^. SNP analysis was performed using the CSI Phylogeny 1.4 server^34^. As the complete and closed chromosome sequences were submitted to the website (https://cge.cbs.dtu.dk/services/CSIPhylogeny/) minimum depth at SNP positions, minimum relative depth at SNP positions, minimum distance between SNPs and minimum SNP quality as input parameters were disabled during analysis. The read mapping quality was set to minimum 25 and the z-score to 1.96. The maximum likelihood tree produced by CSI Phylogeny 1.4 server was visualized in MEGA6 software^35^. *Ad hoc* whole genome multilocus sequence typing (wgMLST) and stable core genome MLST (cgMLST) also called gene-by-gene methods were performed with the Ridom SeqSphere^+^ software (version 3.2.1) using the cgMLST Target Definer tool with the default parameters as previously described^36^. Each allele in the cgMLST approach was assigned a number and an allelic profile called cluster type based on the combination of all alleles was defined for each isolate by the software. Identification of acquired antimicrobial resistance genes was conducted by the ResFinder 2.1 server^37^. To detect a molecular basis of resistance (developing by chromosomal SNPs) against quinolones and rifampicin, *gyrA, gyrB, grlA, grlB* and *rpoB*, respectively, the nucleotide allele sequences were translated with the DNASTAR SeqBuilder software (version 13.0.0) and the amino acid exchanges were identified by alignment using the DNASTAR MegAlign software (version 13.0.0). The *in silico* PFGE banding patterns produced from whole genome sequences were analyzed using BioNumerics software (Applied Maths, Sint-Martens-Latem, Belgium). The definition of a PFGE cluster was based on a similarity cutoff of 80%^38^ using Dice coefficient, represented by the Unweighted Pair Group Method with Arithmetic Mean (UPGMA), 0.5% optimization and 1.0% tolerance.

### Outgroup genomes

To provide a wider context to the data, we utilized 6 fully annotated complete *S. aureus* genomes, epidemiologically unrelated to the Suriname isolates and belonging to the USA300 lineage. The strain names and accession numbers are as follows: UA-S391_USA300, CP007690; USA300_2014.C01 CP012119; USA300_2014.C02, CP012120; USA300_FPR3757, CP000255; USA300_TCH1516, NC_010079; USA300-ISMMS1, CP007690.

### Nucleotide sequence accession numbers

Chromosome (n = 24) and plasmid (n = 63) sequence data of the 24** ***S*. aureus isolates were annotated using the NCBI Prokaryotic Genome Annotation Pipeline and deposited in GenBank under accession numbers CP014362-CP014448.

## Additional Information

**How to cite this article:** Sabat, A. J. *et al*. Complete-genome sequencing elucidates outbreak dynamics of CA-MRSA USA300 (ST8-*spa* t008) in an academic hospital of Paramaribo, Republic of Suriname. *Sci. Rep.* **7**, 41050; doi: 10.1038/srep41050 (2017).

**Publisher's note:** Springer Nature remains neutral with regard to jurisdictional claims in published maps and institutional affiliations.

## Supplementary Material

Supplementary Dataset 1

## Figures and Tables

**Table 1:** Demographic and clinical data of patients infected with MRSA at the AZP in a 2-month period, between 26th of March and 28th of May 2013.

Sample	Isolation date	Patient/Personnel	Age	Body site	Department
SUR1	26-March	Patient-1	<1	Perineum	NICU
SUR2	26-March	Patient-1	<1	Groin	NICU
SUR3	24-April	Patient-2	75	Wound fluid	NNC
SUR4	25-April	Patient-3^+,*,#^	54	Urine	ICU, Neurology, Sanatorium
SUR5	25-April	Patient-3^+,*,#^	54	Sputum	ICU, Neurology, Sanatorium
SUR6	27-April	Patient-4^+^	39	Sputum	ICU
SUR7	30-April	Patient-5	52	Pus leg	Surgery
SUR8	2-May	Patient-6^#^	28	Nose	Sanatorium
SUR9	2-May	Patient-7^#^	71	Nose	Sanatorium
SUR10	3-May	Patient-8^#^	53	Nose	Sanatorium
SUR11	3-May	Patient-8^#^	53	Perineum	Sanatorium
SUR12	9-May	Patient-9	67	Wound left foot	Surgery
SUR13	9-May	Patient-10	44	Pus left axilla	Surgery
SUR14	14-May	Patient-11^*^	65	Wound secretion	Neurology
SUR15	17-May	Patient-9	67	Nose	Surgery
SUR16	17-May	Patient-12^*^	51	Perineum	Neurology
SUR17	21-May	Patient-11^*^	65	Throat	Neurology
SUR18	21-May	Patient-11^*^	65	Nose	Neurology
SUR19	21-May	Patient-11^*^	65	Perineum	Neurology
SUR20	23-May	Nurse^+^	36	Nose	ICU
SUR21	27-May	Patient-13	73	Pus	Emergency room
SUR22	28-May	Nurse^+^	36	Pus axilla	ICU
SUR23	28-May	Nurse^+^	36	Perineum	ICU
SUR24	28-May	Patient-9	67	Perineum	Internal medicine

Superscript indexes indicate patients who stayed in the same ward and room during overlapping periods. Superscript indexes (^+,*,#^) indicate patients/nurse who were in a contact.

**Table 2:** Resistance data.

Antibiotic	ATCC29213 (MIC^a^)	USA 300 isolates from Suriname (MIC^a^)	Acquired gene(s)	Location	Core gene	Substitution
Penicillin	R (0.5)	R (24–48)	*blaZ*	pUSA04		
Oxacillin	S (0.25)	R (>256)	*mecA*	SCC*mec* cassette		
Cefoxitin	S (3)	R (24–128)	*mecA*	SCC*mec* cassette		
Vancomycin	S (0.75)	S (0.50–1)				
Clindamycin	S (0.047)	S (0.047–0.094) and R (>256)^b^	*ermC*	pUSA05		
Linezolid	S (2)	S (0.75–3)				
Rifampicin	S (0.008)	S (0.004–0.012) and R (>32)^c^			*rpoB*	H481Y
Sulfamethoxazole/Trimethoprim	S (0.032)	S (0.0320.75)				
Gentamicin	S (0.38)	S (0.19–0.75)				
Kanamycin	S (3)	R (>256)	*aphA3*	pUSA04		
Streptomycin	S (6)	S (3–12) and R (>1024)^c^	*str*	pUSA06		
Tobramycin	S (0.25)	S (0.25–1)				
Ciprofloxacin	S (0.25)	R (>32)			*grlA*	S80Y
					*gyrA*	S84L
Erythromycin	S (0.5)	R (32- > 256)	*msrA, mphC*	pUSA04		
Mupirocin	S (0.094)	S (0.094–0.25)				
Minocycline	S (0.094)	S (0.047–0.125)				
Tetracycline	S (0.75)	S (0.125–1)				
Bacitracin	R (>256)	R (>256)	*bcrA, bcrB*	pUSA04		
Chloramphenicol	S (4)	S (3–8)				
Daptomycin	S (0.25)	S (0.125–0.75)				
Fusidic Acid	S (0.125)	S (0.032–0.5)				

^a^μg/ml.

^b^Resistance only in two isolates (SUR15 and SUR24) of cluster A.

^c^Resistance only in cluster.
